# Integration of co-culture and transport engineering for enhanced metabolite production

**DOI:** 10.5511/plantbiotechnology.24.0312b

**Published:** 2024-09-25

**Authors:** Yasuyuki Yamada, Miya Urui, Nobukazu Shitan

**Affiliations:** 1 Laboratory of Medicinal Cell Biology, Kobe Pharmaceutical University, Kobe, Hyogo 658-8558, Japan

**Keywords:** co-culture, metabolic engineering, specialized plant metabolites, transport engineering

## Abstract

Microbial production of valuable plant metabolites is feasible. However, constructing all pathways in a single cell is a formidable challenge, and the extended biosynthetic pathways within cells often result in reduced productivity. To address these challenges, a co-culture system that divides biosynthetic pathways into several host cells and co-cultures has been developed. Various combinations of host cells, along with the optimal conditions for each co-culture, have been documented, leading to the successful production of valuable metabolites. In addition, efficient biosynthesis frequently involves metabolite movement, encompassing substrate uptake, intracellular intermediate transport, and end-product efflux. Recent advances in plant transporters of specialized metabolites have enhanced productivity by harnessing these transporters. This review summarizes the latest findings on co-culture systems and transport engineering and provides insights into the future of valuable metabolite production through the integration of co-culture and transport engineering.

## Introduction

Plant metabolites are often used as raw materials in pharmaceuticals and fragrances (Croteau et al. 2000). Therefore, plants and their metabolites are of great importance. However, the growth of most plants is slow, and such bioactive metabolites often accumulate at low levels in specific tissues and organs. Metabolic engineering, also known as synthetic biology, involves the introduction of biosynthetic genes into microorganisms, and has succeeded in producing valuable metabolites (Cravens et al. 2019; Pyne et al. 2019). An early landmark in metabolic engineering was the production of artemisinic acid, a precursor of the antimalarial drug artemisinin (Ro et al. 2006). Thereafter, various metabolites have been successfully produced, including thebaine, an important opiate (Galanie et al. 2015; Nakagawa et al. 2016), cannabinoids, valuable constituents of *Cannabis* (Luo et al. 2019), vinblastine, an anti-cancer compound (Zhang et al. 2022), and tropane alkaloids, neurotransmitter inhibitors (Srinivasan and Smolke 2020, 2021). *E. coli* and *S. cerevisiae* have commonly served as hosts for metabolic engineering due to their ease of genetic manipulation and rapid proliferation (Cravens et al. 2019; Pyne et al. 2019; Yang et al. 2020). In recent years, *Pichia pastoris* (*Komagataella phaffii*) has emerged as a host for metabolic engineering because of its high enzyme expression (Schwarzhans et al. 2017; Shrivastava et al. 2023). Metabolites successfully produced using *P. pastoris* include nootkatone (Wriessnegger et al. 2014), dammarenediol-II (Liu et al. 2015), 6-hydroxydaidzein (Chiang et al. 2016), resveratrol (Kumokita et al. 2022), catharanthine (Gao et al. 2023), naringenin (Kumokita et al. 2022), and reticuline (Kumokita et al. 2022).

However, construction of an entire biosynthetic pathway for a complex metabolite within a single cell is often laborious and time-consuming. Additionally, determining the optimal conditions for all biosynthetic enzymes in a single cell is challenging. To address these issues, a co-culture system has been developed wherein biosynthetic pathways are divided into several modules, each containing partial pathways. Production was achieved through the co-culturing of such modules. In recent years, this co-culture system has demonstrated successful production of various metabolites (Jawed et al. 2019; Thuan et al. 2022).

Substrate uptake, intracellular intermediate movement, and end-product efflux from the cells play crucial roles in metabolite production. Transporters are pivotal for the efficient movement of metabolites across membranes. Advances in transporter studies of specialized metabolites have opened avenues for enhancing productivity through metabolite transport (Belew et al. 2022; Nogia and Pati 2021). This technological approach is recognized as transport engineering.

Co-culture and transport engineering have undergone significant developments. However, for optimal production efficiency, the integration of co-culture systems and transport engineering has the potential to achieve rapid and heightened production of target metabolites. This review summarizes the latest findings of co-culture and transport engineering and discusses the future perspectives of metabolic engineering.

## Co-culture system

**Figure 1:**
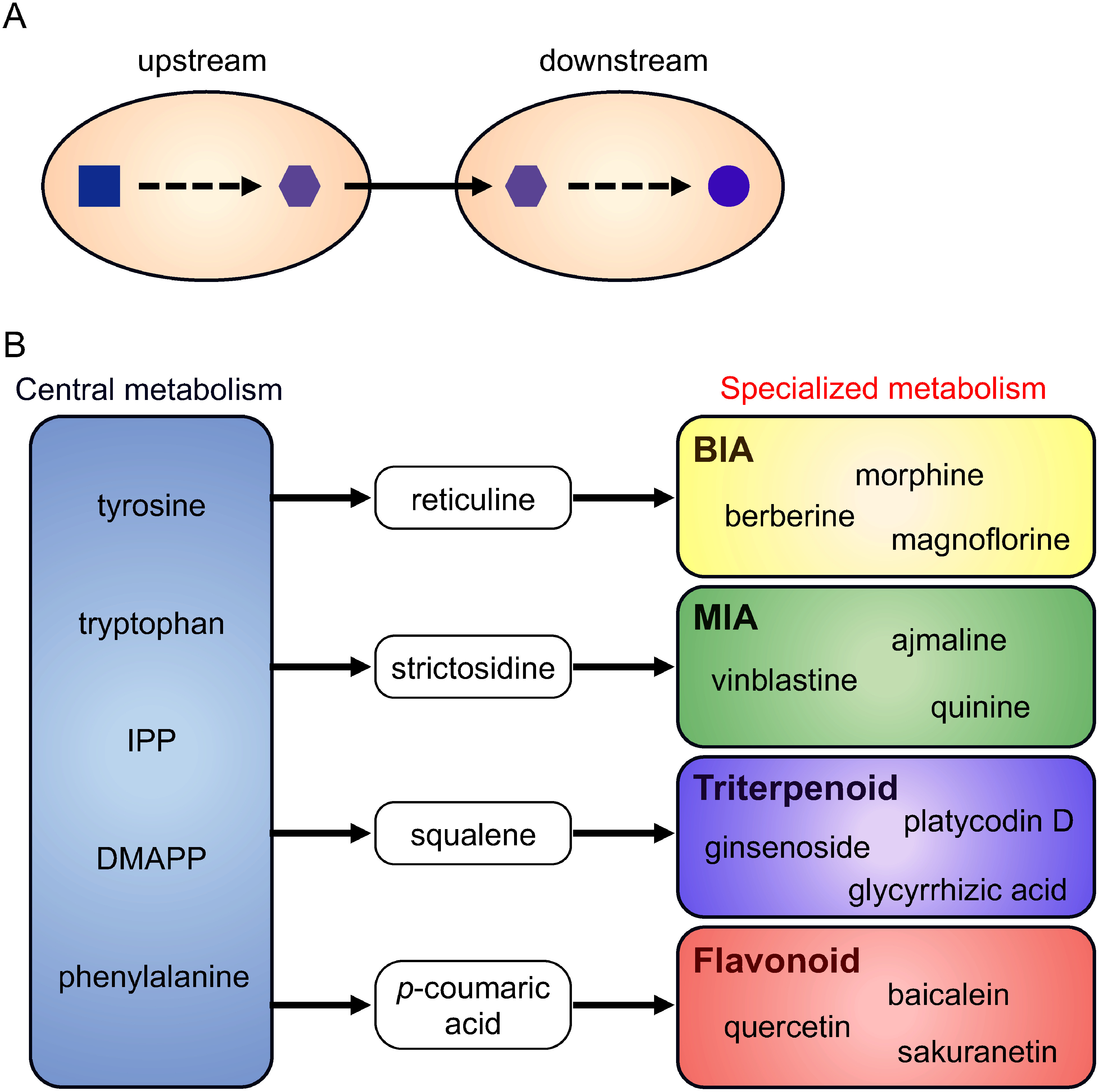
Figure 1. Representative model of co-culture production (A) and the biosynthesis scheme of representative specialized metabolites (B). Abbreviations: BIA, benzylisoquinoline alkaloid; DMAPP, dimethylallyl pyrophosphate; IPP, isopentenyl pyrophosphate; MIA, monoterpene indole alkaloid.

Despite progress in microbial production, the construction of an entire biosynthetic pathway within a single cell remains time-consuming. In addition, productivity is often compromised by several factors. First, metabolic burden is a reported challenge, as the introduction of biosynthetic genes and extended synthetic pathways can impede cell growth and production rates (Wu et al. 2016). Second, the negative feedback or cytotoxicity of intermediates or end products is a recognized issue. Third, the involvement of various enzymes in biosynthesis, including those localized to the ER and vacuoles, poses challenges. Enzymes may exhibit varying functionalities when expressed in different host organisms, such as *E. coli* and *S. cerevisiae*, with instances where an enzyme may function more effectively in one host organism than in another. Providing optimal conditions for all the enzymes within a single cell is challenging. To address these issues, a microbial co-culture system has been developed that involves division of biosynthetic pathways into two or more modules, followed by co-culture (Jawed et al. 2019; Roell et al. 2019; Thuan et al. 2022) (Figure 1). This method can minimize the metabolic burden of synthetic pathways, reduce negative feedback, and provide optimal conditions for each enzyme. For instance, the expression of cytochrome P450 and vacuole-localized enzymes is facilitated in eukaryotic cells such as *S. cerevisiae*. The co-culture system enhanced the production of valuable metabolites. Moreover, many specialized plant metabolites share common intermediates, such as reticuline for benzylisoquinoline alkaloids (BIAs), strictosidine for monoterpene indole alkaloids (MIAs), squalene for triterpenoids, and *p*-coumaric acid for phenylpropanoids (Figure 1B). Consequently, by seamlessly swapping downstream strains in a plug-and-play manner, it becomes feasible to efficiently and conveniently produce target compounds in a short timeframe (Jawed et al. 2019; Roell et al. 2019; Thuan et al. 2022).

Co-culturing the same species is more logistically manageable than co-culturing different species, particularly with respect to culture conditions, such as temperature and medium. The co-culture of *E. coli*-*E. coli* has demonstrated the production of diverse metabolites, including indigo (Chen et al. 2021), phenylpropanoids (Brooks et al. 2023), pinene (Niu et al. 2018), resveratrol (Camacho-Zaragoza et al. 2016; Hong et al. 2020), sakuranetin (Wang et al. 2020), and other metabolites (Thuan et al. 2022). The optimal conditions for these biosynthetic processes have been systematically investigated and established. For instance, phenylpropanoid production was accomplished using a tripartite *E. coli* co-culture (Brooks et al. 2023). The eugenol biosynthetic pathway was divided into three functional modules: module I (from glycerol to *p*-coumaric acid), module II (from *p*-coumaric acid to ferulic acid), and module III (from ferulic acid to eugenol). Eugenol production was observed upon co-culturing of these modules, whereas it was not detected in the monoculture of *E. coli* with the entire eugenol biosynthetic pathway. Through optimization of the initial inoculation ratio of the three strains and the medium composition, elevated eugenol production was achieved. Brooks et al. successfully produced other valuable metabolites, such as hydroxychavicol and chavicol, by strategically swapping downstream strains (Brooks et al. 2023). Co-culture of *S. cerevisiae*-*S. cerevisiae* has also yielded metabolites, such as naringenin and resveratrol (Lu et al. 2021). In addition, *P. pastoris*-*P. pastoris* co-culture has been instrumental in producing stylopine from reticuline (Hori et al. 2016) and lovastatin (Liu et al. 2018).

The co-culture of different species yielded diverse metabolites. *E. coli*-*S. cerevisiae* co-culture demonstrated the production of magnoflorine (Minami et al. 2008), naringenin (Zhang et al. 2017), resveratrol (Yuan et al. 2020), oxygenated isoprenoids such as taxadien-5a-acetate (Zhou et al. 2015), and other metabolites (Thuan et al. 2022). For resveratrol production, the upstream *E. coli* module producing *p*-coumaric acid from glycerol and the downstream *S. cerevisiae* module converting *p*-coumaric acid to resveratrol were co-cultured. Through temperature optimization, adjusting the inoculation ratio of both modules, and optimizing the culture time, the maximum yield reached 36 mg l^−1^ in a test tube (Yuan et al. 2020). Another co-culture combination, *E. coli*-*P. pastoris* has been established for the production of the alkaloid stylopine (Urui et al. 2023, 2021). In this co-culture, the upstream *E. coli* module producing reticuline from glycerol and the downstream *P. pastoris* module converting reticuline to stylopine were co-cultured. For *E. coli*-*P. pastoris* co-culture, the use of BMMY medium, typically employed for *P. pastoris*, and a higher inoculation ratio of *E. coli* compared to *P. pastoris* are recommended for enhanced production. In addition to these combinations, other reported combinations include cadaverine production using *E. coli*-*Corynebacterium glutamicum* co-culture (Sgobba et al. 2018), *O*-methylated phenylpropanoids such as 3,5,4′-trimethoxystilbene production using *E. coli*-*Streptomyces venezuelae* co-culture (Cui et al. 2019), isoprene production using *Synechococcus elongates*-*E. coli* co-culture (Liu et al. 2021), and gamma-glutamylisopropylamide and L-theanine production using *Corynebacterium glutamicum*-*Pseudomonas putida* co-culture (Benninghaus et al. 2024). The synergy between these different species facilitates the production of a wide array of metabolites.

## Transport engineering

The biosynthesis of plant metabolites occurs in various plant organs, tissues, and organelles. Consequently, metabolites, including intermediates and end products, dynamically move among organs, tissues, and organelles (Shitan and Yazaki 2007). Transporters play a crucial role in facilitating these movements, and several transporters have been identified and characterized (Gani et al. 2021; Nogia and Pati 2021; Shitan 2016; Xu et al. 2023): ATP-binding cassette (ABC), multidrug and toxic compound extrusion (MATE), nitrate transporter1/peptide transporter family (NPF), purine uptake permease (PUP), and usually multiple amino acids move in and out transporter (UMAMIT).

In *Nicotiana tabacum*, nicotine is synthesized in the roots, translocated to the aerial parts through the xylem, and finally accumulates in leaf vacuoles (Baldwin 1989). Nicotine transport involves specific MATE transporters, such as NtJAT1 (jasmonate-inducible alkaloid transporter1) and NtMATE1, along with the PUP transporter NtNUP1 (Hildreth et al. 2011; Kato et al. 2015; Morita et al. 2009; Shitan et al. 2015, 2014; Shoji et al. 2009).

In *Catharanthus roseus*, vinblastine biosynthesis occurs across three leaf tissues: internal phloem parenchyma, epidermis, and laticifers (Kulagina et al. 2022). NPF transporters, namely, NPF2.4, NPF2.5, NPF2.6, and NPF2.9, participate in the inter- and intracellular movement of vinblastine intermediates (Kulagina et al. 2022; Larsen et al. 2017; Payne et al. 2017).

In opium poppy, opioids, such as codeine and morphine, are biosynthesized and accumulated. The early biosynthetic steps from norcoclaurine to salutaridine or thebaine occur in sieve elements. Salutaridine and/or thebaine then are transported to the laticifer, where those are converted to codeine and morphine (Ozber and Facchini 2022). Among the identified BIA uptake permeases (BUPs) in the biosynthetic gene clusters, BUP1 would be implicated in the salutaridine and/or thebaine uptake in laticifer cells (Dastmalchi et al. 2019).

Advancements in transport studies have paved the way for utilizing transport engineering for microbial production (Belew et al. 2022; Lv et al. 2016; Nogia and Pati 2021). This includes substrate uptake from the medium, efficient movement of intermediates across vacuolar membranes, and efflux of end products from cells, thereby enhancing productivity, as elaborated below.

**Figure 2:**
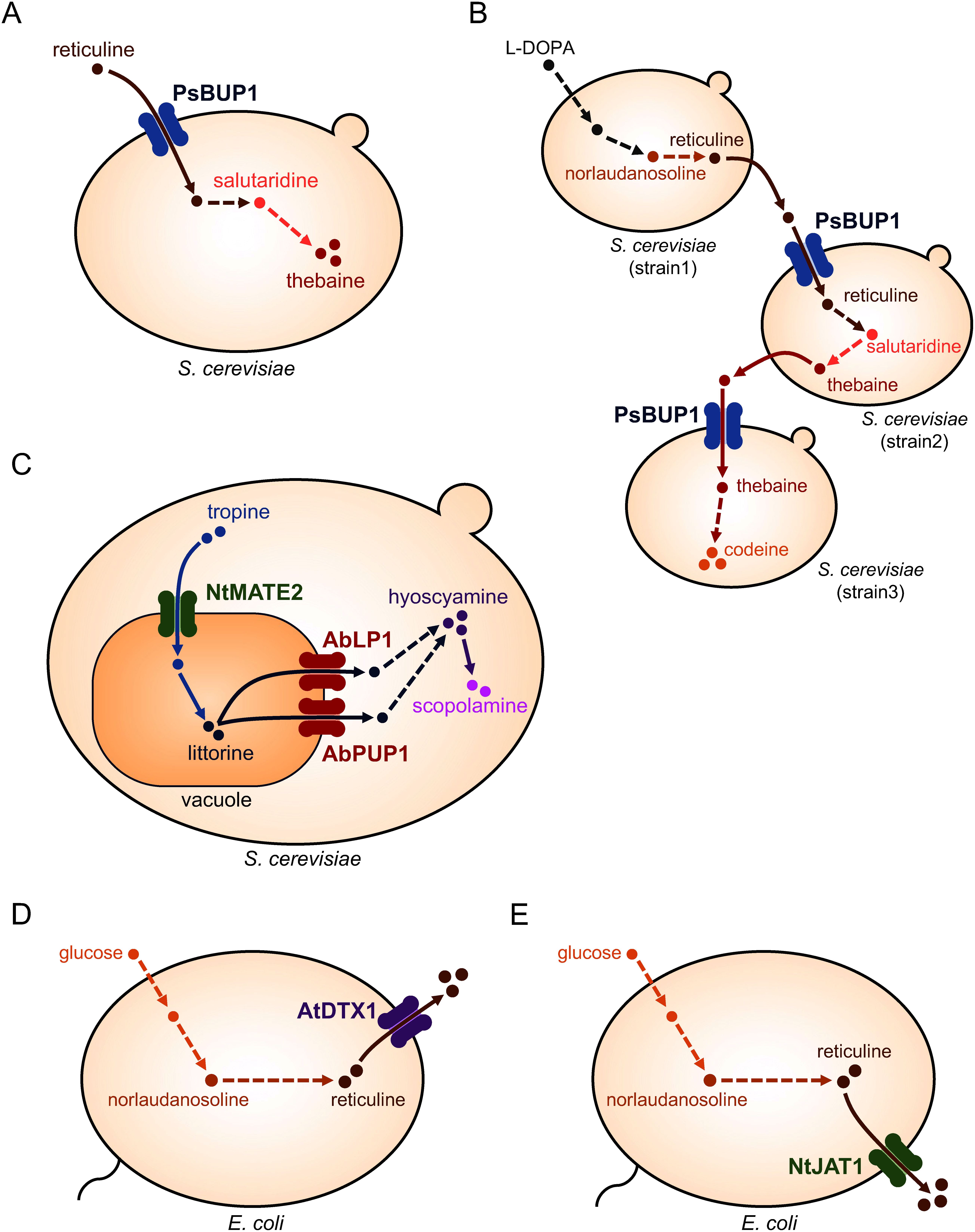
Figure 2. Representative transport engineering models. (A) Enhancement of thebaine production through increased reticuline import facilitated by opium poppy BUP1. (B) Enhanced codeine production through modular co-culture and BUP1 expression. (C) Enhanced tropane alkaloid production through expression of NtMATE2, AbPUP1, and AbLP1. (D) Enhanced reticuline production via AtDTX1 expression. (E) Enhanced reticuline production via NtJAT1 expression. Transporter names: AbLP1, *Atropa belladonna* lactose permease-like transporter1; AbPUP1, *Atropa belladonna* purine uptake permease1; AtDTX1, *Arabidopsis thaliana* detoxification1; NtJAT1, *Nicotiana tabacum* jasmonate-inducible alkaloid transporter1; NtMATE2, *Nicotiana tabacum* multidrug and toxic compound extrusion2; PsBUP1, *Papaver somniferum* BIA uptake permease1.

Substrate uptake transporters play a crucial role in enhancing opioid biosynthesis. The expression of BUP1 in *S. cerevisiae*, which produces thebaine from reticuline, significantly increased thebaine production (5-fold) (Figure 2A). Additionally, BUP1 expression in a co-culture of three *S. cerevisiae* strains, each with a partial opiate biosynthesis pathway from L-DOPA, enhanced the productivity of thebaine and codeine (Figure 2B) (Dastmalchi et al. 2019).

Intermediate transport amplifies the production of tropane alkaloids such as hyoscyamine and scopolamine in *S. cerevisiae*. Owing to the localization of acyltransferase (littorine synthase) to the vacuole in the biosynthesis of littorine, an essential intermediate from tropine and phenyllactic acid glucoside, transporters were introduced to facilitate intermediate movement. Expression of the tobacco MATE transporters NtJAT1 and NtMATE2, which possibly transport tropine into the vacuole, significantly enhanced tropane alkaloid production (Srinivasan and Smolke 2020). Introducing other transporters, *Atropa belladonna* PUP1 and lactose permease-like transporter (LP1), likely efflux littorine from the vacuole to the cytosol, coupled with NtMATE2 expression and optimization of conditions, improved hyoscyamine and scopolamine production by 100-fold and 7-fold, respectively (Srinivasan and Smolke 2021) (Figure 2C).

The export of end products also enhances biosynthesis, because some metabolites may be toxic to microorganisms and contribute to negative feedback. Expression of the Arabidopsis MATE transporter detoxification1 (AtDTX1) in *E. coli*, which produces reticuline from glycerol, improved reticuline production 11-fold (Yamada et al. 2021) (Figure 2D). AtDTX1 expression upregulated several internal metabolic pathways, especially those related to reticuline biosynthesis, such as pentose-phosphate and methionine biosynthetic pathways. Similarly, expression of the tobacco MATE transporter NtJAT1 in reticuline-producing *E. coli* improved production by 14-fold (Yamada et al. 2022) (Figure 2E). A comparable increase in production via end-product efflux has been reported in phlorizin biosynthesis. The expression of Arabidopsis AtPUP8 in phlorizin-producing *S. cerevisiae* improved biosynthesis by 1.8-fold (Belew et al. 2020). These examples demonstrate the use of plant transporters in transport engineering.

Engineering using transporters from non-plant organisms has also been reported (Belew et al. 2022; Lv et al. 2016; Nogia and Pati 2021). The expression of microbial efflux transporters in amorphadiene-producing *E. coli* cells improves amorphadiene production by more than three-fold (Zhang et al. 2016). Similarly, the expression of vacuolar-localized bile pigment transporter1 (BPT1) from *S. cerevisiae*, along with other optimizations, increased cannabidiol production 100-fold in *S. cerevisiae* cells (Qiu et al. 2022). BPT1 also increased glycyrrhetinic acid production in *S. cerevisiae* (Alkhadrawi et al. 2022), demonstrating the feasibility of transport engineering using transporters isolated from non-plant organisms. However, transporting intermediates with structures similar to those of the target compounds may lead to decreased productivity. Therefore, transporters with a narrow substrate specificity may be required. Identifying such transporters from plants that produce target metabolites and their application could be beneficial, as exemplified by the enhanced production of tropane alkaloids using AbPUP1 isolated from *A. belladonna* (Srinivasan and Smolke 2021) (Figure 2C). Further identification and characterization of plant transporters of specialized metabolites are crucial for advancing these studies.

Additionally, directed evolution (Arnold 2018; Wang et al. 2021) offers a promising approach to enhance transporter activity and productivity, as demonstrated by the directed evolution of transporters for enhanced 3-hydroxypropionate production (Liang et al. 2023). Moreover, recent advancements in structural analyses (Baril et al. 2023) and structural predictions using deep learning, such as Alphafold2 (Janes and Beltrao 2024), have enabled the rational design of transporters with specific substrate specificity. For instance, mutations predicted based on structural analysis altered the substrate specificity of SWEET13, a transporter for both sucrose and gibberellin (Isoda et al. 2022). These innovations will further improve productivity and advance transport engineering.

## Concluding remarks and future perspectives

Microbial co-culture and transport engineering have enabled the production of diverse valuable metabolites and enhanced productivity. However, these two technologies have often been independently investigated. Two examples of combining co-culture and transport engineering for metabolite production are noteworthy. In the case of codeine production, the expression of BUP1 for substrate uptake was pivotal for culturing three *S. cerevisiae* cells, resulting in improved productivity (Dastmalchi et al. 2019) (Figure 2B). Another example involves co-culture for 4-hydroxystyrene production, where upstream *E. coli* producing tyrosine and downstream *E. coli* producing 4-hydroxystyrene from tyrosine are co-cultured. Introducing a tyrosine efflux transporter into the upstream strain facilitated tyrosine efflux, enhancing 4-hydroxystyrene productivity (Gargatte et al. 2021). In co-culture, efficient cell-to-cell movement of intermediates is crucial; hence, the expression of efflux and influx transporters in the upstream and downstream modules, respectively, could enhance productivity, resembling a “push-and-pull strategy” for efficient nitrogen allocation and nutrient improvement in plants (Yadav et al. 2015). For instance, a co-culture of reticuline-producing *E. coli* cells expressing AtDTX1 (Yamada et al. 2021) and thebaine-producing *S. cerevisiae* cells expressing BUP1 (Dastmalchi et al. 2019) may be advantageous for higher thebaine production from glycerol. Furthermore, the additional expression of an efflux transporter for the end product would also allow efficient recovery of the target compound from the culture medium.

**Figure 3:**
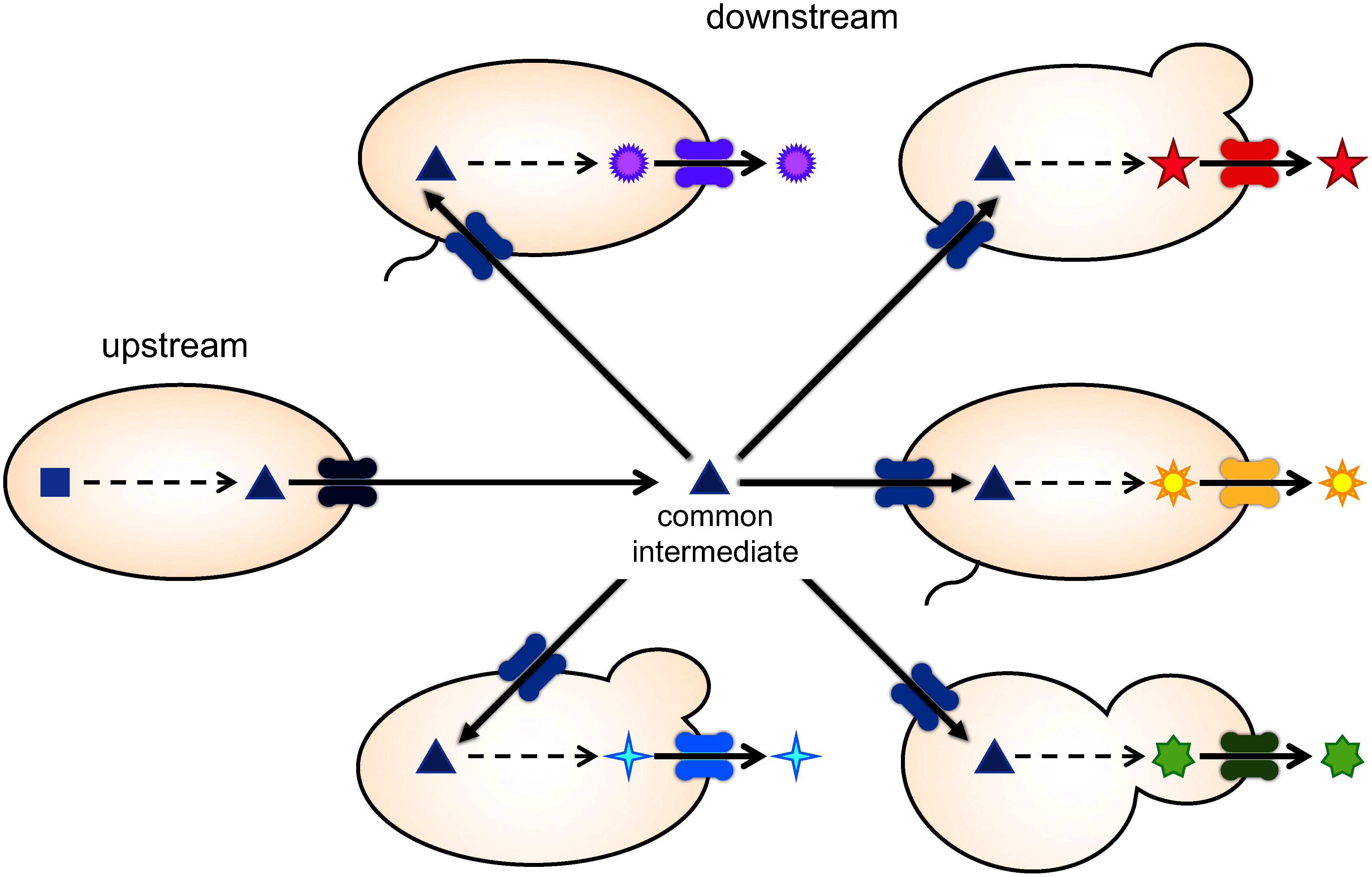
Figure 3. Production of valuable metabolites through the integration of co-culture and transport engineering. Integration of “plug-and-play” and “push-and-pull” strategies will lead to the efficient production of various metabolites.

Specialized plant metabolites are synthesized through various modifications of common intermediates derived from the central metabolism (Figure 1B). Combining plug-and-play and push-and-pull strategies facilitates rapid and efficient production of target metabolites (Figure 3). In addition, novel metabolites can be generated by combining different modules for metabolite modification. Various combinations of co-culture and transport engineering hold promise for producing novel pharmaceutical materials. Further development of these technologies is expected to enhance their impact on human health.

## Abbreviations

ABC: ATP-binding cassette
BIA: benzylisoquinoline alkaloid
BPT1: bile pigment transporter1
BUP: BIA uptake permease
DMAPP: dimethylallyl pyrophosphate
DTX1: detoxification1
ER: endoplasmic reticulum
IPP: isopentenyl pyrophosphate
LP: lactose permease-like transporter
MATE: multidrug and toxic compound extrusion
MIA: monoterpene indole alkaloid
NPF: nitrate transporter1/peptide transporter family
PUP: purine uptake permease
UMAMIT: usually multiple amino acids move in and out transporter
